# A targeted performance improvement programme reduces mortality in exsanguinating pelvic trauma

**DOI:** 10.1186/1757-7241-21-S1-S28

**Published:** 2013-05-28

**Authors:** ZB Perkins, GD Maytham, L Koers, P Bates, K Brohi, NRM Tai

**Affiliations:** 1 Trauma Sciences, Queen Mary, University of London, London, UK; 2 Trauma Clinical Academic Unit, The Royal London Hospital, Whitechapel, London, UK; 3 Academic Department of Military Surgery and Trauma, Royal Centre for Defence Medicine, Birmingham, UK

## Background

Shocked patients with pelvic fractures are amongst the most severely injured patients requiring trauma care. They present a complex management challenge with mortality rates of up to 60%, the majority being the result of early exsanguination.[1] Errors and delays in the process of care contribute to this substantial mortality. Multidiscipline performance improvement programmes (PIP) aim to monitor the quality of care and identify opportunities for improvements that may affect outcome. They are a critical component of verified US trauma centres.[2] Our aim was to examine the impact of a targeted PIP on outcome in shocked patients with pelvic fractures.

## Methods

We analysed the clinical care and PIP activities of all adult patients presenting to our Major Trauma Centre with exsanguinating pelvic trauma, for a period of 4 years (2007 – 2010) following the incorporation of the American College of Surgeons Trauma PIP[3] into local governance processes. Multivariable logistic regression adjustment for baseline variables was performed and outcome trends were compared with national data.

## Results

One hundred eighty-five shocked patients with pelvic fractures were included. Median Injury Severity Score was 34 and median base deficit was 8.25 mEq/L. Sixty-two patients (34%) died from their injuries. The PIP identified pitfalls in the structure or process of care in one third of deaths. The most common were ‘errors in decision-making leading to delays or inappropriate treatment’ (32%) and ‘delays in blood or blood product availability’ (27%). Implemented changes included: 1) a decision-making algorithm for the management of exsanguinating pelvic trauma, 2) a massive haemorrhage protocol and 3) appointment of specialist pelvic orthopaedic surgeons. These changes were associated with significant annual improvements in the targeted processes of care, including: time to massive haemorrhage protocol activation (p=0.04); volume of crystalloid transfused (p<0.01); ratios of blood to blood-products transfused (p<0.0001); time to primary surgical haemorrhage control (p=0.05) and proportion of patients undergoing definitive pelvic fixation (p=0.01). Survival improved from 45% (2007) to 79% (2010) with a significant annual reduction in mortality (p=0.002) that remained after adjusting for baseline patient and injury characteristics. This trend was not evident nationally.

## Conclusion

The institution of a PIP allowed the identification and targeted improvement of aspects of trauma care that impact outcome and resulted in rapid and sustained mortality reductions in this severely injured patient group.
